# Differences in metaviromes between *Aedes aegypti* and *Aedes albopictus* from sympatric areas on Hainan Island and the Leizhou Peninsula, China

**DOI:** 10.1186/s13071-026-07371-8

**Published:** 2026-03-26

**Authors:** Minghui Zhao, Yuting Jiang, Xin Ran, Yun Liao, Qiang Zhang, Yu Bai, Qian Liu, Tongyan Zhao, Hengduan Zhang

**Affiliations:** 1 State Key Laboratory of Pathogen and Biosecurity, Academy of Military Medical Sciences, Beijing, China; 2 Jiangxi International Travel Healthcare Center, Nanchang, China; 3 Jiangxi Provincial Center for Disease Control and Prevention, Nanchang, China

**Keywords:** *Aedes aegypti *, *Aedes Albopictus*, Metavirome

## Abstract

**Background:**

*Aedes aegypti* and *Ae. albopictus* are the most important vector mosquito species globally and are capable of transmitting various viral diseases, such as dengue fever, zika virus disease, and chikungunya fever. Although they overlap in terms of ecological niches and geographical distribution, their virus carriage and transmission capacities differ significantly. Metavirome studies can provide new perspectives for understanding these differences.

**Methods:**

In this study, next-generation sequencing (NGS) was used to analyze the epidemiologically significant metaviromes of *Ae. aegypti* and *Ae. albopictus* on Hainan Island and the Leizhou Peninsula, China. A bioinformatics analysis pipeline was used to compare the viral compositions of the two mosquito species.

**Results:**

In the sympatric areas, 250 viral species from 60 families were annotated to *Ae. aegypti* at the read level, whereas 406 vial species from 66 families were annotated to *Ae. albopictus* at the read level, revealing significant differences in the metaviromes of the two mosquito species. Notably, *Ae. albopictus* exhibited significantly greater viral diversity than *Ae. aegypti* (*p* < 0.05). The 50 viruses with the greatest abundance in two mosquito species were selected for data analysis, revealing 64% viral similarity, with 32 common viruses and 18 distinct viruses between the two species, although the relative abundances of each virus differed notably. Phasi Charoen-like Phasivirus (PCLV) from *Phenuiviridae* showed the highest relative abundance in all *Ae. aegypti* sample pools, whereas *Orthophasmavirus barstukasense* (*Phasmaviridae*), *Gihfavirus pelohabitans* (*Steitzviridae*), and unclassified Wenzhou sobemo-like virus 4 (WSLV4) predominated in different *Ae. albopictus* sample pools.

**Conclusions:**

The metavirome compositions of *Ae. aegypti* and *Ae. albopictus* in the sympatric areas of Hainan Island and the Leizhou Peninsula differed significantly. The viral diversity of *Ae. albopictus* was significantly higher than that of *Ae. aegypti*, and notable differences in viral composition and abundance were observed between the two species. However, the 50 most abundant viruses detected in both mosquito species also exhibited a degree of similarity. These findings support further research into the viral compositions of these two *Aedes* species. Moreover, analyzing these distinct viral compositions aids understanding of the vector capacity and vector competence of these mosquitoes, which will provide theoretical support for vector control efforts on Hainan Island and the Leizhou Peninsula.

**Graphical Abstract:**

**Figure:**
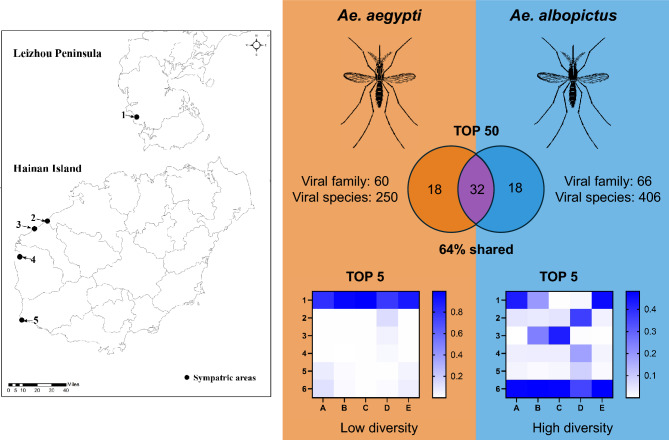

**Supplementary Information:**

The online version contains supplementary material available at 10.1186/s13071-026-07371-8.

## Background

*Ae. aegypti* and *Ae. albopictus* are well known as vectors of infectious diseases such as yellow fever, dengue fever, chikungunya fever, and zika virus disease, which cause significant economic losses and burdens in tropical, subtropical, and temperate regions each year [1–3]. In recent years, the development of next-generation sequencing (NGS) technology has enabled the discovery of many pathogenic viruses in these mosquitoes. Moreover, an increasing number of nonpathogenic viruses have been identified, some of which are closely related to vector competence. The number of reports on the mosquito virome has increased from 2 publications in the early 2000s to more than 200 in recent years. These reports span at least 128 species of mosquitoes across 14 genera, and more than 380 viruses have been identified in the *Ae. aegypti* population alone. The vast majority of these are insect-specific viruses (ISVs) [4–6]. For example, studies of the metaviromes of *Ae. aegypti* and *Ae. albopictus* in Geneva have identified numerous nonpathogenic viruses belonging to various families [7]; research into the metaviromes of male mosquitoes in southwestern Brazil has revealed a significant number of ISVs [8]; and studies of the metaviromes of *Ae. albopictus* in southern Switzerland’s Ticino Canton have identified 13 viral species, including nonpathogenic insect-specific flaviviruses, which were present in all sample pools [9].

Research on the metaviromes of *Ae. aegypti* and *Ae. albopictus* has indicated that the “core viromes” of both mosquito species are composed mainly of ISVs, including *Flaviviridae*, *Totiviridae*, *Phenuiviridae*, *Orthomyxoviridae*, *Virgaviridae*, and *Secoviridae*, which affect the susceptibility of mosquitoes to certain arthropod-borne viruses and are related to the evolution of these viruses [10–12]. Additionally, studies have reported significant differences in the richness, diversity, and abundance of “core viromes” between the two mosquito species [13]. The abundance of ISVs in *Ae. aegypti* is relatively high, which is related to their high susceptibility to pathogen infections, while the diversity of ISVs in *Ae. albopictus* is greater, possibly because of their different life cycles or ecological and environmental factors [14]. The “core viromes” of *Ae. albopictus* can be further divided into “vertically transmitted” and “environmental source” core viromes [15]. In addition to the “core viromes,” a “core microbiome” is present in the mosquito genome. Other studies have shown that the core microbiota of *Ae. aegypti* and *Ae. albopictus* interact with arthropod-borne viruses to modulate the susceptibility of vector mosquitoes. For example, the transmission efficiency of dengue virus (DENV), zika virus (ZIKV), and chikungunya virus (CHIKV) is significantly reduced in *Aedes* mosquitoes infected with *Wolbachia* [16–18]. Additionally, research has shown that laboratory-reared *Ae. aegypti* and *Ae. albopictus* share similar gut microbiota compositions, while the composition of the whole-body microbiota differs significantly, indicating that the gut microbiota is determined by the environment, whereas the whole-body microbiota is strongly influenced by genetics [19].

Extensive research into the mosquito metagenome has also been conducted across various regions of China. A metavirome study was conducted on mosquitoes collected from different habitats in Guangdong Province, China, identifying 101 virus species and finding that the viral composition is closely related to host species, with *Culex* showing greater viral diversity than *Aedes* [20]. Another study revealed that the viral composition of *Ae. albopictus* in Guangzhou is mainly seasonal; human parechovirus and hepatitis B virus were first discovered in *Ae. Albopictus* [21]. A metavirome study on a mixed group of mosquitoes collected from Yunnan Province, including *Ae. aegypti* and *Ae. albopictus*, identified viruses from 22 viral families, including CHIKV, Getah virus, and Ross River virus, with CHIKV first isolated from *Culex tritaeniorhynchus* [22, 23]. A metavirome study on mosquitoes collected from the Shanxi–Gansu–Ningxia region revealed 116 viruses from 31 viral families, further revealing that the viral diversity of *Culex* is greater than that of *Aedes* and *Anopheles* and that viral diversity and abundance are also influenced by collection time [24]. A metavirome study on mosquitoes in Hubei Province revealed two new viruses from the family *Densovirinae* and the family *Dicistroviridae* family [25]. A metavirome study on mosquitoes collected in Shanghai identified viruses from 48 viral families and revealed that *Banna virus* can cause human encephalitis [26]. In 2018, metavirome sequencing of *Ae. aegypti* on the Leizhou Peninsula revealed PCLV, which belongs to the family *Phenuiviridae* and the genus *Phasivirus,*, to be widely present in the *Ae. aegypti* population in that region [27]. From 2018 to 2020, a metavirome study on mosquitoes on Hainan Island revealed 57 known viruses from 15 families and 39 new viruses, indicating that most RNA viruses persist in different locations and different mosquito species on Hainan Island, demonstrating the stability of the mosquito virome on Hainan Island [28]. However, owing to the small sample size of *Ae. aegypti*, only one virus was found in its sample group, which was not reflective of the virome characteristics of *Ae. aegypti* on Hainan Island. This study aims to investigate the metaviromes of sympatric *Ae. aegypti* and *Ae. albopictus* on Hainan Island and the Leizhou Peninsula, to compare the viral profiles of the two mosquito species and to identify potential pathogenic viruses and ISVs that may influence vector competence and disease transmission. These findings will contribute to understanding of the complex interactions between mosquitoes and their viral communities, with implications for arbovirus surveillance and control strategies in those regions.

## Methods

### Mosquito collection

**Fig. 1:**
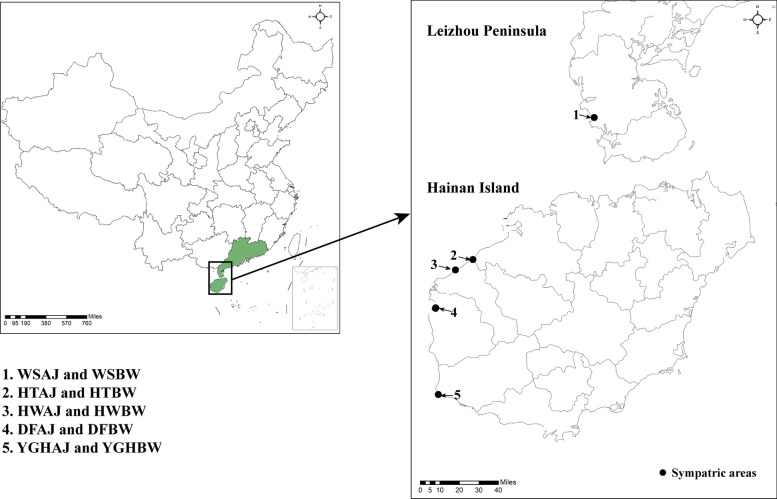
Sampling map of *Ae. aegypti* and *Ae. albopictus* populations from sympatric fields

In July 2023, mosquito samples were collected from the sympatric areas of *Ae. aegypti* and *Ae. albopictus* on the Leizhou Peninsula and Hainan Island. The WS population of *Ae. aegypti* was collected in July 2021 because *Ae. aegypti* was not found in that area in 2023 [29]. Sampling information, location, and population code are presented in Table 1 and Fig. 1. *Aedes* larvae were collected using the larval suction method. Adult *Aedes* mosquitoes in breeding containers were captured by an electric aspirator (catalog no. DX3V, CHICO·J·X), and those in their surrounding environments were captured. These *Ae. aegypti* and *Ae. albopictus* were sourced from the same positive breeding containers, such as tires, foam boxes with standing water, and flowerpots with standing water. The distance between these positive containers did not exceed 1000 m, and at least five positive container larvae were mixed into one sampling pool for subsequent analysis. The collected adult mosquitoes were also within a 1000-m range. All adult mosquitoes were morphologically identified after emergence [30]. After collection, any adult individuals with visibly undigested blood in their abdomens were excluded, and the remaining were then maintained on sugar solution for 24 h, allowing sufficient time for any residual blood in the abdomen to be fully digested. Female mosquitoes from each sampling point were selected as a sample pool (10–40 females per pools as shown in Table 1) and stored at −80°C for future use.

**Table 1:** Collection information of *Ae. aegypti* and *Ae. albopictus* populations from sympatric fields

Mosquito	Collection region	Locality	Code	Total (female)	Longitude/latitude
*Ae. aegypti*	Leizhou	Wushizhen	WSAJ	10	109° 50′ 36″ E, 20° 33′ 13″ N
	Hainan	Haitouzhen	HTAJ	40	108° 56′ 54″ E, 19° 30′ 34″ N
		Haiweizhen	HWAJ	23	108° 49′ 07″ E, 19° 25′ 56″ N
		Dongfang	DFAJ	17	108° 40′ 25″ E, 19° 09′ 09″ N
		Yinggehai	YGHAJ	17	108° 41′ 29″ E, 18° 30′ 30″ N
*Ae. albopictus*	Leizhou	Wushizhen	WSBW	40	109° 50′ 54″ E, 20° 33′ 43″ N
	Hainan	Haitouzhen	HTBW	38	108° 56′ 54″ E, 19° 30′ 34″ N
		Haiweizhen	HWBW	37	108° 49′ 07″ E, 19° 25′ 56″ N
		Dongfang	DFBW	19	108° 40′ 25″ E, 19° 09′ 09″ N
		Yinggehai	YGHBW	23	108° 41′ 29″ E, 18° 30′ 30″ N

### RNA extraction

Mosquitoes stored at −80 °C were removed and placed into grinding tubes (cat. no. WMG010, Shanghaijingxin). Six to eight grinding beads (cat. no. YG0113, Shanghaijingxin) were added to each grinding tube, followed by the addition of 600 µl of physiological saline. After the lid was tightly sealed, the tubes were placed in a homogenizer for 45 s (5000 rpm, a low speed to prevent the generation of excessive heat and mechanical shearing, as these could degrade viral RNA). After centrifugation, the supernatant was collected for nucleic acid extraction, following the instructions of the QIAamp Viral RNA Mini Kit (#52,904). DNase treatment to deplete genomic DNA from the RNA extracts was performed using a Turbo DNA-free kit (AM1907, Thermo Fisher Scientific) following the manufacturer’s instructions. The extracted RNA was quantified using a Qubit 4 Fluorometer and an RNA HS Assay Kit (Q32855, Invitrogen) following the manufacturer’s protocol. Samples were only used for library construction if they had an RNA concentration of ≥ 20 ng/μL (Additional file 1: Table S1).

### Library construction and sequencing

First, viral RNA enrichment was performed using the pathogen RNA enrichment platform (BGI Genomics). Then, ribosomal RNA was removed using a method based on streptavidin magnetic beads to specifically capture rRNA sequences.

Second, fragmentation, reverse transcription, and library construction were performed. The Covaris instrument was used for ultrasonic fragmentation. The fragmented sample was then subjected to fragment selection, which concentrates the sample band around 200–400 bp. Reverse transcription using random hexamer primers (six-base random oligonucleotides N_6_, where N represents a random mixture of A, T, C, and G nucleotides at each position) was used to synthesize the first strand of complementary DNA (cDNA). The complementary strand was synthesized concurrently with end repair and A-tailing to yield the second strand of cDNA. A polymerase chain reaction (PCR) system was used for amplification (95 °C for 3 min; 15 cycles of: denaturation: 95 °C for 30 s, annealing: 60 °C for 30 s, extension: 72 °C for 30 s; final extension: 72 °C for 5 min, hold: 4 °C), and the amplified PCR products were subjected to library quality control.

Finally, the MGISEQ-2000 was used for sequencing. Qualified products were further circularized into single-stranded products; linear DNA molecules that failed to circularize were digested. Single-stranded circular DNA molecules underwent rolling circle amplification to form a DNA nanosphere containing multiple copies. The obtained DNA nanospheres were added to the mesh holes on the chip (MGISEQ-2000 RS Flow Cell, a high-density nanoarray chip designed to load DNA nanospheres into its mesh holes for high-throughput sequencing) and sequenced through a combined probe anchoring polymerization technique. These adaptor-specific, fluorescently labeled oligonucleotide probes hybridize to the adaptor regions of the DNA Nanoballs, initiating the polymerization-based sequencing reaction. On the MGISEQ-2000 platform, PE150 sequencing (150 bp paired-end) was performed using dual-indexing for multiplex library pooling. Each library is projected to yield approximately 10 million reads.

### Data analysis

First, the raw sequencing data were processed to obtain clean data (https://www.bioinformatics.babraham.ac.uk/projects/trim galore/). The specific steps were as follows: first, reads containing 10% uncertain bases (N bases) were removed; second, reads containing sequencing adapter sequences were removed (regions of 15 bases or longer aligned to the adapter sequence); and finally, reads containing more than 40% low-quality bases (bases with Q < 20) were removed. Next, to reduce the interference of host sequences on subsequent analyses, Bowtie2 (https://bowtie-bio.sourceforge.net/bowtie2/manual.shtml) was used to filter host information from *Ae. aegypti* and *Ae. albopictus*, after which sequences aligned to the genomes of *Ae. aegypti* (GCF 002204515.2) and *Ae. albopictus* (GCF 006496715.2) were removed [31]. Finally, the Kraken method (http://ccb.jhu.edu/software/kraken/) was used for comparison [32], and species annotation at the read level was performed in the National Center for Biotechnology Information (NCBI) nt database. Viral abundance was calculated on the basis of the number of reads obtained through sequencing. The relative abundance (%) in our study was calculated using the following formula: (number of annotated reads for a specific contig/total number of annotated reads) × 100. ggplot2 plotting analysis was conducted on the R platform [33, 34]. The heatmap visualization is presented in ComplexHeatmap [35].

## Results

### Sampling and sequencing results

A total of five field populations of *Ae. aegypti* and five field populations of *Ae. albopictus* were collected and combined into ten sample pools (Table 1). These pools were then subjected to viromic sequencing, yielding a total of 2.10 × 10^9^ reads and 3.12 × 10^11^ bases. The Q30 was above 80%, with an average of 87.64% (Additional file 1: Table S2).

### Virus diversity and host analysis

**Fig. 2:**
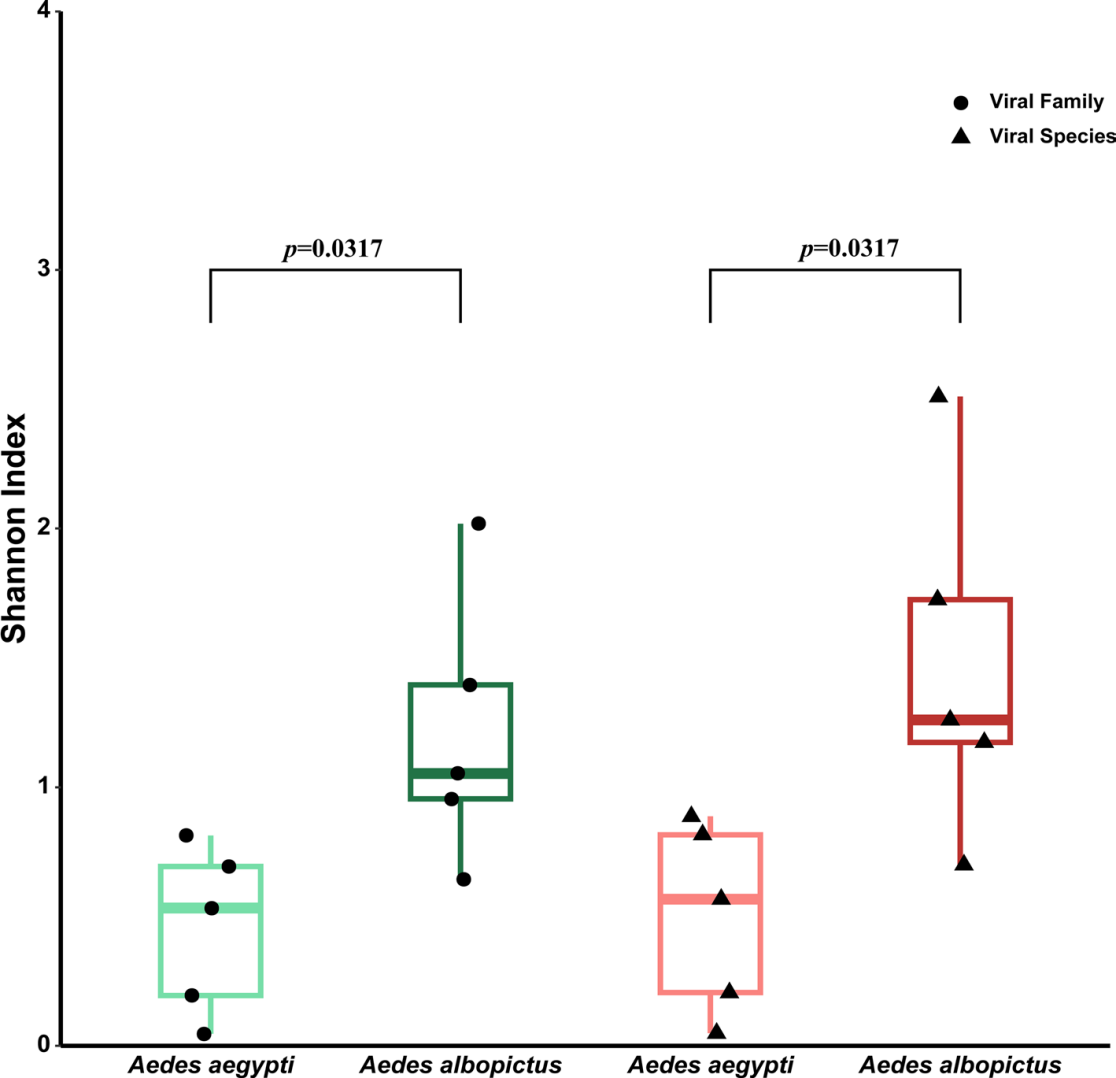
The *α* diversity of viruses in the *Ae. aegypti* and *Ae. albopictus* pools from different areas at different taxonomic levels

**Fig. 3:**
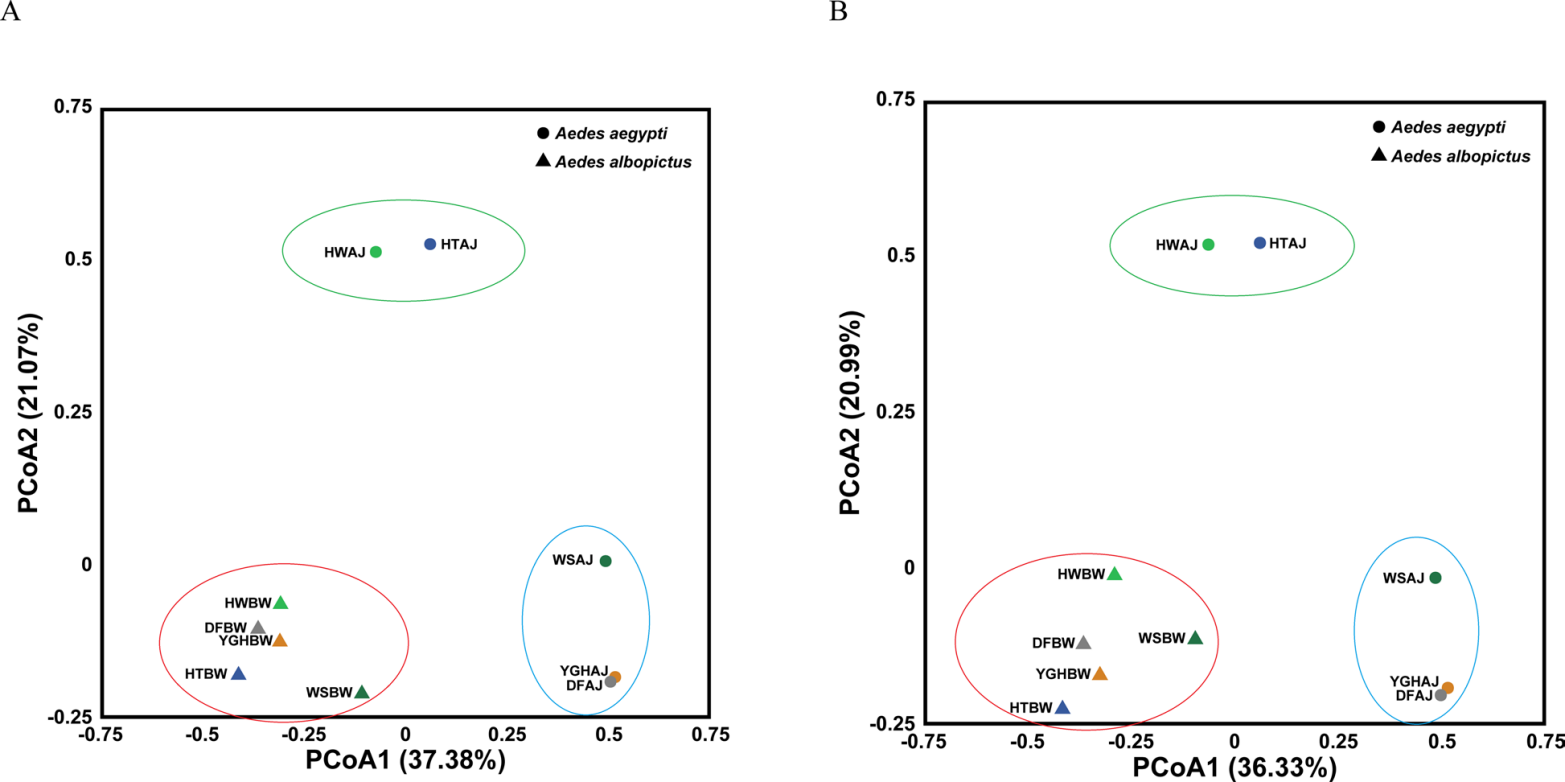
The *β* diversity of viruses in the *Ae. aegypti* and *Ae. albopictus* pools at **A** family level and **B** species level; the red circle represents one branch of *Ae. albopictus*, while the blue and green circles represent two branches of *Ae. aegypti*

A total of 250 known viruses from 60 families and 154 genera were annotated at the read level from *Ae. aegypti*, while 406 known viruses from 66 families and 228 genera were annotated from *Ae. albopictus* (Additional file 1: Table S3)*.* Then, *α*- and *β*-diversity for *Ae. aegypti* and *Ae. albopictus* at different taxonomic levels were constructed on the basis of the variation in viral diversity across the sample pools (Figs. 2 and 3). Significant differences (*p* = 0.0317 < 0.05) in *α*-diversity between the *Ae. aegypti* and *Ae. albopictus* sample pools at the family and species levels are shown in Fig. 2. The Shannon diversity index in *Ae. albopictus* was significantly greater than that in *Ae. aegypti*. The significant differences in *β*-diversity at the family and species levels are shown in Fig. 3. The five *Ae. albopictus* pools formed a distinct cluster. The other five *Ae. aegypti* pools formed two branches: the HWAJ and HTAJ pools clustered together, while the remaining three pools clustered separately.

**Fig. 4:**
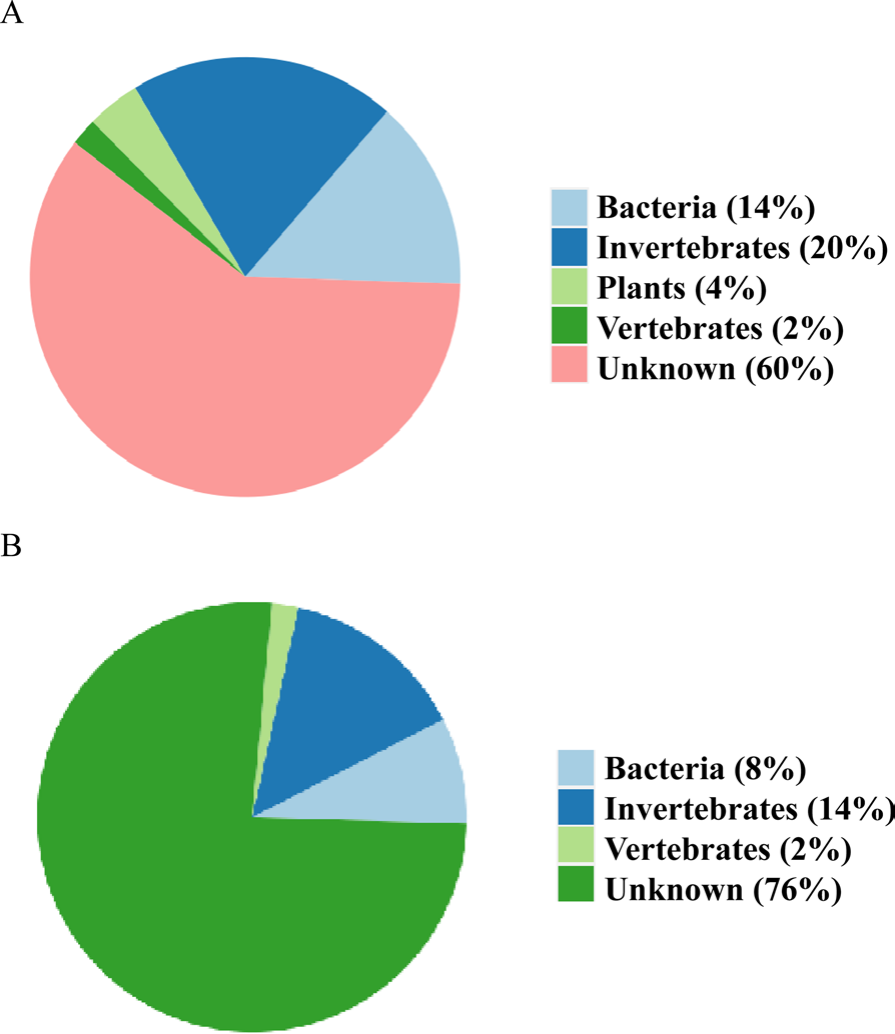
Composition of the hosts of the top 50 viruses in the *Ae. aegypti* (**A**) and *Ae. albopictus* (**B**) pools

To better analyze the viral composition of the two mosquito species, we selected the 50 viruses with the greatest relative abundance for subsequent data analysis (Additional file 4: Table S4). The host sources of the 50 most abundant viruses in *Ae. aegypti* include 7 types of bacteria, 1 type of vertebrate virus, 10 types of invertebrate viruses, 2 types of plant viruses, and 30 types of viruses with undetermined host sources (Fig. 4A). The host sources of the 50 most abundant viruses in *Ae. albopictus* included 4 types of bacteria, 1 type of vertebrate virus, 7 types of invertebrate viruses, and 38 types of viruses with undetermined host sources (Fig. 4B).

The 50 most abundant viruses in *Ae. aegypti* and *Ae. albopictus* could be divided into 22 known virus families and unclassified viruses; among these, 15 viral families were present in both mosquito species. At the family level, the 22 viral families of *Ae. aegypti* included *Tobaniviridae*, *Straboviridae*, *Steitzviridae*, *Sedoreoviridae*, *Poxviridae*, *Potyviridae*, *Polydnaviriformidae*, *Phycodnaviridae*, *Phenuiviridae*, *Phasmaviridae*, *Peribunyaviridae*, *Peduoviridae*, *Mitoviridae*, *Mimiviridae*, *Intestiviridae*, *Inoviridae*, *Herelleviridae*, *Hantaviridae*, *Flaviviridae*, Baculoviridae, *Autographiviridae*, and *Alphatetraviridae*, for a total of 30 viruses. *Phenuiviridae* exhibited the greatest relative abundance in all sample pools. The remaining 20 viruses were unclassified viruses. The 22 viral families of *Ae. albopictus* included *Zierdtviridae*, *Tospoviridae*, *Tobaniviridae*, *Straboviridae*, *Steitzviridae*, *Rhabdoviridae*, *Polydnaviriformidae*, *Phenuiviridae*, *Phasmaviridae*, *Peribunyaviridae*, *Peduoviridae*, *Mitoviridae*, *Mimiviridae*, *Leisingerviridae*, *Kyanoviridae*, *Inoviridae*, *Herpesviridae*, *Hantaviridae*, *Chaseviridae*, *Baculoviridae*, *Autographiviridae*, and *Ascoviridae*, with a total of 36 viruses. *Phasmaviridae*, *Steitzviridae*, and unclassified viruses had the highest relative abundance. The remaining 14 viruses were unclassified viruses.

At the species level, the 50 most abundant viruses in *Ae. aegypti* and *Ae. albopictus* were highly consistent, with 32 viruses (64%) shared between the two species. The top 10 viruses were markedly similar, with only one unique virus each [cell fusing agent virus (CFAV) in *Ae. aegypti* and *Orthophasmavirus barstukasense* in *Ae. albopictus*]; the remaining nine viruses were common to both mosquitoes (PCLV, *Gihfavirus pelohabitans*, *Dhakavirus bp7*, *Escherichia virus DE3*, *Kinglevirus lutadaptatum*, *Simbu orthobunyavirus*, *Inovirus M13*, *Teseptimavirus T7*, and *Biseptimavirus P1105*). CFAV (*Flaviviridae*) was detected only in the five *Ae. aegypti* pools and not in any of the *Ae. albopictus* pools. However, *Orthophasmavirus barstukasense* was also prevalent in all five *Ae. aegypti* pools.

### Relative abundance

**Fig. 5:**
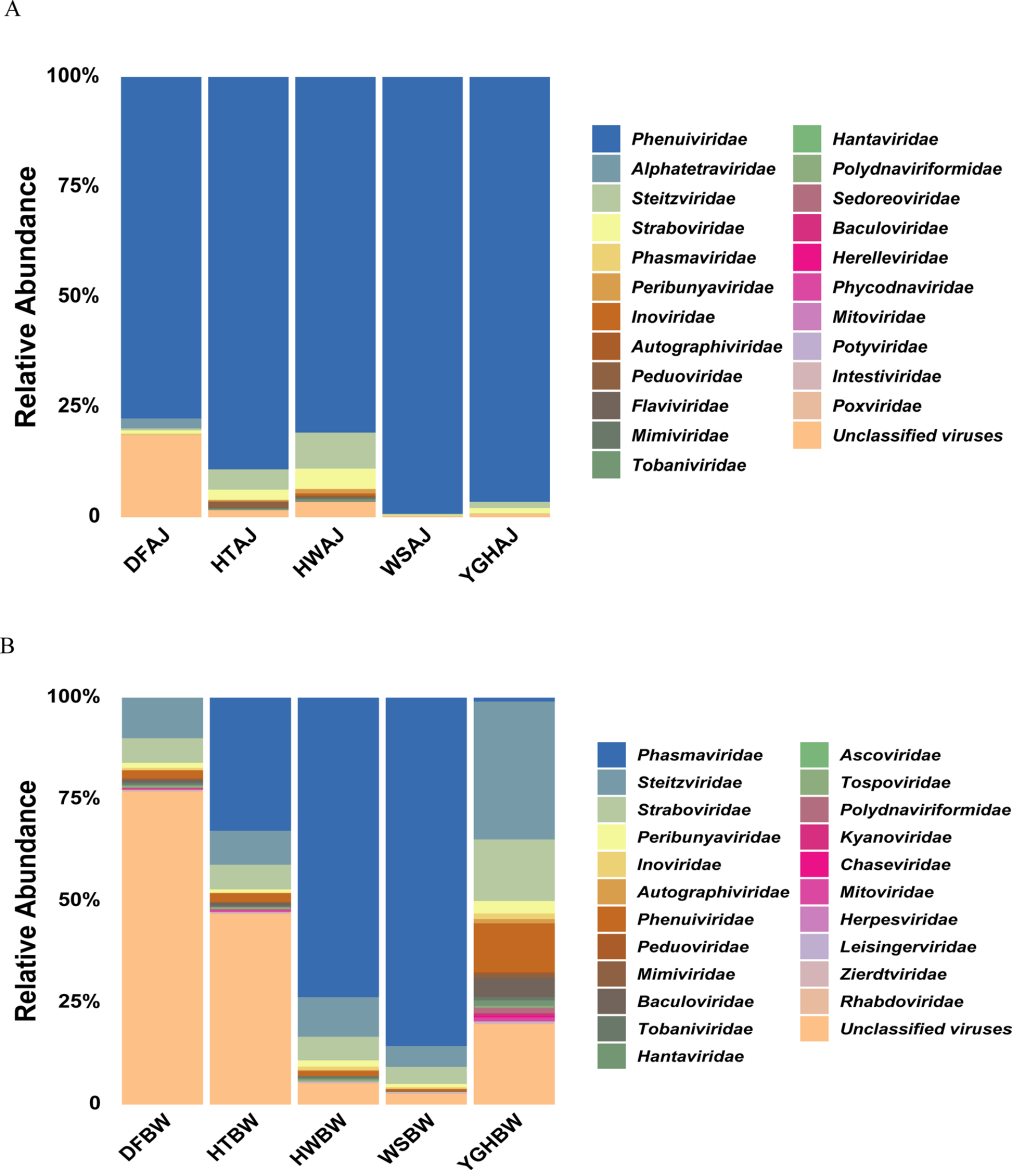
Relative abundance of viral families in the *Ae. aegypti* (**A**) and *Ae. albopictus* (**B**) pools

**Fig. 6:**
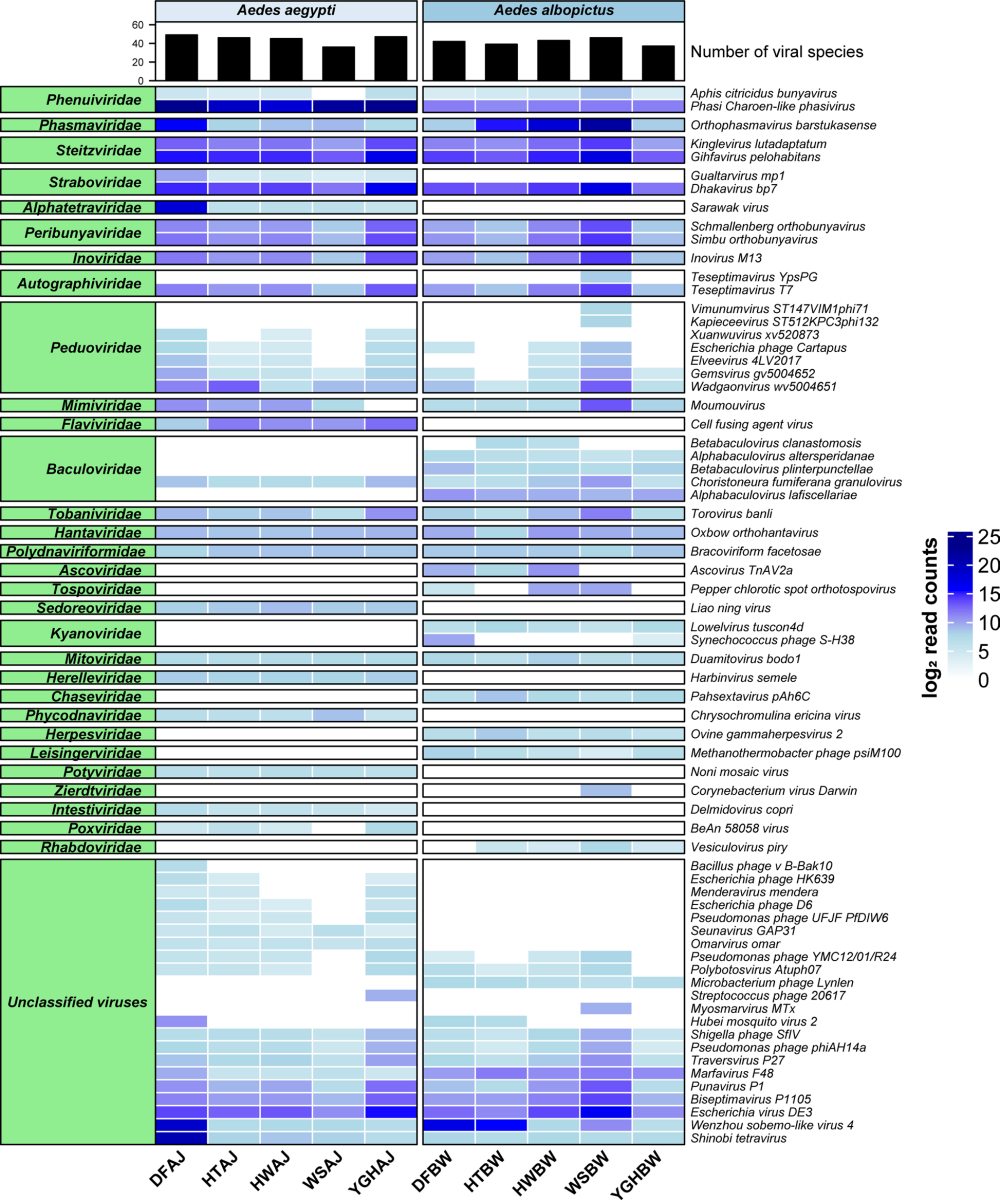
Heatmap of the top 50 viruses in the *Ae. aegypti* and *Ae. albopictus* pools (viral species in at least one pool). The bar plot above shows the number of viral species in each pool

Relative abundance plots (Fig. 5) and heatmaps (Fig. 6) of the top 50 viruses were generated for *Ae. aegypti* and *Ae. albopictus* on the basis of the virus reads analyzed. The *Phenuiviridae* family exhibited the greatest relative abundance across all five *Ae. aegypti* sample pools, demonstrating absolute dominance. The WSAJ sample pool displayed the highest relative abundance (99.43%), while the DFAJ sample pool presented the lowest (77.34%). Conversely, unclassified viruses exhibited the highest relative abundance in the DFAJ pool (18.50%) and the lowest in the WSAJ sample pool (0.08%). The DFAJ pool exhibited the greatest number of species within the top 50 viruses (49 viruses excluding *Streptococcus phage* 20,617). In contrast, the WSAJ pool had the fewest species among the top 50 viruses (36 viruses). The data in Fig. 5B reveal significant differences in viral relative abundance across the different *Ae. albopictus* sample pools. Viruses in *Phasmaviridae* family were most abundant in the HWBW and WSBW pools (73.82% and 85.74%, respectively), whereas unclassified viruses were most abundant in the DFBW and HTBW pools (77.12% and 47.09%, respectively). *Steitzviridae* viruses were most abundant in the YGHBW pool (33.89%). In the WSBW pool, 46 species among the top 50 viruses were detected, and in the YGHBW pool, only 37 species were detected.

As shown in Fig. 6, 32 viruses were present in both *Aedes* species. Of these, 20 belonged to known viral families, whereas 12 remained unclassified. Among those 20 viruses, 14 were detected in all the sample pools. Four of these viruses, namely PCLV, *Kinglevirus lutadaptatum*, *Gihfavirus pelohabitans*, and *Dhakavirus bp7*, exhibited high relative abundances across all ten sample pools. Nine of the 12 unclassified viruses were detected across all sample groups, with *Biseptimavirus P1105* and *Escherichia virus DE3* exhibiting high abundances in ten sample pools. Among those six highly abundant viruses, only PCLV (*Phenuiviridae*) was an ISV; the other five were unclassified. Additionally, 18 viruses were detected only in *Ae. aegypti,*, and 18 were detected only in *Ae. albopictus*. In *Ae. aegypti*, *Gualtarivirus mp1*, CFAV, *Sarawak virus*, *Lianoning virus*, and *Chrysochromulina ericina virus* were highly abundant across all sample pools. In *Ae. albopictus*, *Betabaculovirus plinterpunctellae*, *Alphabaculovirus lafiscellariae*, *Pahsextavirus pAh6C* and, *Ovine gammaherpesvirus 2* exhibited high abundances across sample pools.

## Discussion

In this study, the metaviromes of *Ae. aegypti* and *Ae. albopictus* in sympatric areas on Hainan Island and the Leizhou Peninsula were compared. Significant differences in metavirome composition were detected between the two species (*p* < 0.05) [36, 37]. Overall, the viral diversity in the *Ae. albopictus* population in the sympatric areas was greater than that in the *Ae. aegypti* population (Fig. 2). These findings are consistent with those of Gómez's study [14]. Further research has found that *Ae. albopictus* in Pakhtunkhwa Province, Pakistan, carry a significantly greater variety of bacterial species than *Ae. Aegypti* [38]. Nevertheless, 32 out of the 50 most abundant viruses were found in both *Ae. aegypti* and *Ae. albopictus*, accounting for 64% of all viruses. The abundances of nine out of the ten most abundant viruses were also consistent. This finding suggests a high degree of similarity in the prevalent viral community structure between the two mosquito species. This similarity may be due to their overlapping ecological niches, similar host preferences, or shared viral evolutionary history [39]. Furthermore, this consistency suggests that interventions targeting these shared viruses could be effective against both species, optimizing disease control strategies [40]. However, the specific mechanisms that lead to this consistency in the prevalent viral community remain unclear. Future research needs to explore virus–host interactions in depth to elucidate the underlying biological basis.

PCLV, belonging to the *Phenuiviridae* family, was most prevalent in *Ae. aegypti*, exceeding a relative abundance of 75% across all sample pools. However, within the *Ae. albopictus* pools, the abundance of this virus was relatively high only in the YGHBW pool (11.50% < 25%) and was low in all the other pools (Figs. 5 and 6). The *Phenuiviridae* family now belongs to the order *Hareavirales*, which comprises 24 genera, including *Bandavirus* and *Phasivirus*. The newly identified severe fever with thrombocytopenia syndrome bunyavirus (SFTSV) is a *Bandavirus* and a significant arbovirus. It causes severe fever with thrombocytopenia syndrome, an acute infectious disease prevalent in Asia [41, 42]. However, PCLV is an ISV belonging to the *Phasivirus* family and is reported to be the most abundant in *Ae. aegypti* worldwide [43, 44]. A report indicated that the virus was first detected in *Ae. aegypti* populations in Wushi on the Leizhou Peninsula in 2018. It was also confirmed that this ISV can be transmitted vertically [27]. Furthermore, high abundance and vertical transmission characteristics of PCLV have been observed in *Ae. aegypti* populations in India [45]. In the absence of alternative hosts or poor environmental persistence, vertical transmission is necessary for the persistence of viruses [46, 47]. In this study, PCLV was also identified to have a relative abundance of up to 99.43% in the WSAJ sample pool (Fig. 5). The high relative abundance of PCLV in the WSAJ sample pool underscores its potential dominance in the local *Ae. aegypti* virome, which may reflect selective environmental or host factors that favor its persistence. Another study revealed that the probability of wild *Ae. aegypti* carrying DENV increases by 200% when PCLV coexists with another ISV, Humaita–Tubiacanga virus (HTV) [44]. Laboratory mouse models verified that the presence of PCLV and HTV enhanced the ability of mosquitoes to transmit DENV and ZIKV to vertebrate hosts [44]. The prevalence of PCLV among *Ae. aegypti* infected with CHIKV was as high as 79.15% in Brazilian populations of this species [48]. However, other studies have shown that PCLV does not affect the infection or replication of mosquito-borne viruses, such as DENV and ZIKV, in *Ae. aegypti* populations [49]. These contrasting findings provide new insights into how ISVs influence the replication of mosquito-borne viruses.

The *Phasmaviridae* and *Steitzviridae* families exhibited the highest detection rates in *Ae. albopictus*. Within the *Phasmaviridae* family, *Orthophasmavirus barstukasense* was relatively abundant at high levels in the HTBW, HWBW, and WSBW sample pools (32.91%, 73.82%, and 85.84%, respectively), whereas within the *Steitzviridae* family, *Gihfavirus pelohabitans* was relatively abundant at high levels in the YGHBW group (29.83%). Viruses from these two families were also present in all the *Ae. aegypti* sample pools, but at a very low relative abundance (all below 7.20%). The *Phasmaviridae* family belongs to the order *Elliovirales* and comprises many ISVs. Members of this family have been found in laboratory-reared *Ae. albopictus* samples from Hubei Province, wild-caught *Ae. albopictus* samples from Guangzhou, and *Culex* samples from Shanxi, Gansu, and Ningxia [15, 20, 24]. The detection of endogenous viral elements (EVEs) in *Ae. aegypti* from different countries worldwide also revealed insertions belonging to the *Phasmaviridae* family [50, 51]. The widespread occurrence of *Phasmaviridae* across geographically distinct regions among those two *Aedes* species underscores its potential as a ubiquitous component of mosquito-associated viromes. The identification of EVEs related to *Phasmaviridae* in *Ae. aegypti* genomes further suggests ancient viral integration events, which may have evolutionary implications for host immune adaptation or viral persistence. In the latest published “Summary of Taxonomic Changes Approved by the International Committee on Taxonomy of Viruses (ICTV),” *Orthophasmavirus barstukasense* was renamed *Orthophasmavirus barstukorius* (BARV) [52]; the revised name derives from the Lithuanian Barstukas, a mythical dragon-like creature, and -orius, for “pertaining to” (ICTV). Further characterization of its replication mechanisms and host range restrictions may yield insights into the coevolutionary dynamics between BARV and its mosquito hosts. The *Steitzviridae* family is the largest family of viruses within the *Timlovirales* order and represents a group of soil bacteriophages [53, 54]. Its high abundance in the YGHBW sample pool (Fig. 5) suggests that it may be an environmentally derived virus. However, the reasons for its elevated abundance in this *Ae. albopictus* sample pool warrant further investigation.

Unclassified viruses account for a high proportion of both the DFBW and the HTBW sample pools. The abundance and diversity of unclassified viruses represent the bulk of the planet’s undescribed genetic diversity and are key to understanding viral evolution, host ranges, ecological distributions, and origins. They allow us to revisit the origin of viruses and their place in the evolutionary history of life [55, 56]. Wenzhou sobemo-like virus 4 (WSLV4) was the dominant unclassified virus in the two *Ae. albopictus* pools (72.48% and 40.90%, respectively), and it was also found in the *Ae. aegypti* pools, with the highest abundance occurring in the DFAJ pool (4.76%). WSLV4 is an ISV and is widely prevalent in *Ae. aegypti* and *Ae. albopictus* populations [9, 15, 57–60]. It has also been reported in other mosquito species, such as *Cx. tritaeniorhynchus* in Shandong Province [61]. WSLV4 was detected in both male and female mosquito sample pools [62], indicating that it can be transmitted vertically [28, 63]. Further studies on WSLV4 in these mosquitoes are needed to better understand the role of this species in the dynamics of arbovirus transmission.

Although the majority of the viruses detected in these two *Aedes* mosquitoes were nonpathogenic, their presence and diversity contribute to understanding the virome composition and potential virus–host interactions in *Aedes* mosquitoes. The most medically important arboviruses belong to *Flaviviridae* (e.g., yellow fever virus, DENV, and ZIKV), *Togaviridae* (e.g., CHIKV and Eastern equine encephalitis virus), and *Bunyaviricetes* (e.g., SFTSV and Orthohantavirus) [64, 65]. These viral families are of significant medical importance, and extensive research has been conducted into them. This study did not detect pathogenic viruses, but CFAV from the *Flaviviridae* family was identified in all the *Ae. aegypti* sample pools. CFAV is an insect-specific flavivirus whose vertical transmission is high and occurs through transovarial transmission in naturally infected *Ae. Aegypti* [66, 67], being widely present in *Ae. albopictus* in Ticino, Switzerland [9]. Further research has confirmed that CFAV can influence the susceptibility of mosquitoes to arboviruses in both in vivo and in vitro coinfection models [68]. Although no pathogenic viruses were detected in this study, ISVs such as PCLV and CFAV may influence the transmission of arboviruses, making them valuable subjects for research. The aim of our study is to broadly characterize the virome of *Ae. aegypti* and *Ae. albopictus*, exploring the ecological and epidemiological significance of their entire viral communities. These findings have broadened understanding of the range of viruses present in *Aedes* mosquitoes and provide a foundation for further research into their functions.

Although this study allowed for extensive screening of viral diversity within *Ae. aegypti* and *Ae. albopictus*, notable limitations that require refinement still exist. First, short-read sequencing technology makes it difficult to accurately assemble complex viral genomes, which has a particular impact on variant analysis. Second, the lack of standardization in bioinformatics analysis workflows and incomplete databases restricted the annotation and functional prediction of highly variable or novel viruses. Furthermore, this method can be used to detect only the presence of viruses, rather than directly assessing the vector capacity, vector competence, or viral biological properties of the virus. Subsequent traditional virus isolation and experimental validation are needed to clarify the ecological and epidemiological significance of the findings.

## Conclusions

In the sympatric areas, *Ae. aegypti* was annotated to 60 families and 250 species of viruses at the read level; *Ae. albopictus* was annotated to 66 families and 406 species of viruses at the read level, revealing significant differences in the metavirome of the two mosquito species, with *Ae. albopictus* showing significantly higher viral diversity than *Ae. aegypti*. The top 50 viruses in terms of abundance revealed 64% virus similarity, with 32 common core viruses and 18 distinct core viruses between the two species, although the relative abundance of each virus differed notably. PCLV from *Phenuiviridae* was most abundant in all the *Ae. aegypti* sample pools, whereas *Orthophasmavirus barstukasense* (*Phasmaviridae*), *Gihfavirus pelohabitans* (*Steitzviridae*), and unclassified virus WSLV4 predominated in the different *Ae. albopictus* sample pools. Understanding these findings will facilitate further research into the viral compositions of these two *Aedes* species. Analyzing their distinct viral compositions aids our understanding of their vector capacity and vector competence, which will provide theoretical support for vector control efforts on Hainan Island and the Leizhou Peninsula.

## Supplementary Information

Additional file 1. Table S1. Quality control results for RNA from *Ae. aegypti* and *Ae. *albopictus pools. Table S2. Summary of the data output for the *Ae. aegypti* and *Ae. albopictus *pools. Table S3. Viral composition at the read level of the *Ae. aegypti *and *Ae. albopictus* pools. Table S4. Viral composition and read numbers of “core viruses” in the *Ae. aegypti* and *Ae. albopictus *pools

## Data Availability

Data supporting the main conclusions of this study are included in the manuscript. The raw sequencing data were deposited in the National Library of Medicine (NIH)-DFBW: SRR36492669, HTBW: SRR36496816, HWBW: SRR36514581, WSBW: SRR36515554, YGHBW: SRR36516174, DFAJ: SRR36537302, HTAJ: SRR36539933, HWAJ: SRR36544822, WSAJ: SRR36496088, YGHAJ: SRR36545336.

## Abbreviations

BARV: *Orthophasmavirus barstukorius*
CFAV: Cell fusing agent virus
CHIKV: Chikungunya virus
DENV: Dengue virus
EVEs: Endogenous viral elements
HTV: Humaita–Tubiacanga virus
ICTV: International Committee on Taxonomy of Viruses
NGS: Next-generation sequencing
ISVs: Insect-specific viruses
PCLV: Phasi Charoen-like Phasivirus
SFTSV: Severe fever with thrombocytopenia syndrome bunyavirus
WSLV4: Wenzhou sobemo-like virus 4
ZIKV: Zika virus
