# Supplementary material for: Differences in metaviromes between Aedes aegypti and Aedes albopictus from sympatric areas on Hainan Island and the Leizhou Peninsula, China
Source: Parasit Vectors. 2026 Mar 26;19:200. doi: 10.1186/s13071-026-07371-8 (PMC13147869; doi:10.1186/s13071-026-07371-8)
Supplement: Supplementary file 1 — Additional file 1. Table S1. Quality control results for RNA from Ae. aegypti and Ae. albopictus pools. Table S2. Summary of the data output for the Ae. aegypti and Ae. albopictus pools. Table S3. Viral composition at the read level of the Ae. aegypti and Ae. albopictus pools. Table S4. Viral composition and read numbers of “core viruses” in the Ae. aegypti and Ae. albopictus pools [file 13071_2026_7371_MOESM1_ESM.doc]

Table S1 Quality control results for RNA from *Ae. aegypti* and *Ae. albopictus* pools

| Populations Code | Concentration(ng/μL) | Volume (μL) |
| --- | --- | --- |
| WSBW | 375 | 40 |
| HTBW | 145 | 40 |
| HWBW | 570 | 40 |
| DFBW | 470 | 40 |
| YGHBW | 130 | 40 |
| WSAJ | 63 | 40 |
| HTAJ | 310 | 40 |
| HWAJ | 645 | 40 |
| DFAJ | 202 | 40 |
| YGHAJ | 535 | 40 |

Table S2 Data output summary of *Ae. aegypti* and *Ae. albopictus* pools

| Mosquito | Code | Sequencing | Reads | Base（bp） | GC（%） | Q30（%） |
| --- | --- | --- | --- | --- | --- | --- |
| *Ae.* | WSAJ | PE150 | 211,388,556 | 31,331,797,866 | 44.22 | 87.62 |
| *aegypti* | HTAJ | PE150 | 211,410,380 | 31,432,502,486 | 43.29 | 87.59 |
|  | DFAJ | PE150 | 211,418,620 | 31,454,222,672 | 43.41 | 87.21 |
|  | HWAJ | PE150 | 211,412,518 | 31,419,672,808 | 43.06 | 87.07 |
|  | YGHAJ | PE150 | 211,410,678 | 31,500,990,076 | 43.25 | 87.73 |
| *Ae.* | WSBW | PE150 | 200,251,590 | 29,785,820,236 | 46.74 | 89.08 |
| *albopictus* | HTBW | PE150 | 211,409,180 | 31,181,067,432 | 45.26 | 89.19 |
|  | DFBW | PE150 | 211,406,276 | 31,458,553,848 | 44.35 | 86.51 |
|  | HWBW | PE150 | 211,429,694 | 31,505,647,522 | 43.67 | 86.96 |
|  | YGHBW | PE150 | 211,410,880 | 31,415,159,562 | 43.88 | 87.44 |
| Total/Average |  |  | 2,102,948,372 | 312,485,434,508 | 44.11 | 87.64 |

Table S3 Virus composition at reads level of *Ae. aegypti* and *Ae. albopictus* pools

| Populations Code | Family | Genus | Species |
| --- | --- | --- | --- |
| HWAJ | 38 | 76 | 124 |
| YGHAJ | 42 | 90 | 140 |
| WSAJ | 37 | 63 | 95 |
| DFAJ | 47 | 95 | 155 |
| HTAJ | 38 | 68 | 102 |
| Total | 60 | 154 | 250 |
| HWBW | 49 | 106 | 169 |
| HTBW | 47 | 117 | 210 |
| DFBW | 45 | 80 | 135 |
| YGHBW | 40 | 82 | 139 |
| WSBW | 51 | 148 | 245 |
| Total | 66 | 228 | 406 |

Table S4 Virus composition and read numbers of the top 50 viruses in *Ae. aegypti* and *Ae. albopictus* pools

| **Family** | **Species** | **HWAJ** | **YGHAJ** | **WSAJ** | **DFAJ** | **HTAJ** |
| --- | --- | --- | --- | --- | --- | --- |
| *Alphatetraviridae* | *Sarawak* virus | 79 | 41 | 71 | 277093 | 71 |
| *Autographiviridae* | *Teseptimavirus* T7 | 1733 | 8223 | 321 | 2792 | 1307 |
| *Baculoviridae* | *Choristoneura* fumiferana granulovirus | 155 | 495 | 137 | 362 | 137 |
| *Flaviviridae* | *Cell* fusing agent virus | 1758 | 4713 | 1416 | 276 | 3107 |
| *Hantaviridae* | *Oxbow* orthohantavirus | 358 | 571 | 447 | 486 | 352 |
| *Herelleviridae* | *Harbinvirus* semele | 237 | 276 | 225 | 283 | 238 |
| *Inoviridae* | *Inovirus* M13 | 1998 | 9585 | 312 | 3313 | 1336 |
| *Intestiviridae* | *Delmidovirus* copri | 57 | 18 | 45 | 122 | 45 |
| *Mimiviridae* | *Moumouvirus* | 1091 | 0 | 167 | 1726 | 809 |
| *Mitoviridae* | *Duamitovirus* bodo1 | 130 | 138 | 154 | 134 | 129 |
| *Peduoviridae* | *Wadgaonvirus* wv5004651 | 75 | 431 | 465 | 2473 | 7195 |
| *Peduoviridae* | *Gemsvirus* gv5004652 | 52 | 252 | 20 | 797 | 48 |
| *Peduoviridae* | *Elveevirus* 4LV2017 | 31 | 129 | 0 | 380 | 20 |
| *Peduoviridae* | *Escherichia* phage Cartapus | 18 | 113 | 0 | 209 | 11 |
| *Peduoviridae* | *Xuanwuvirus* xv520873 | 13 | 42 | 0 | 184 | 0 |
| *Peribunyaviridae* | *Simbu* orthobunyavirus | 2381 | 11592 | 493 | 3992 | 1643 |
| *Peribunyaviridae* | *Schmallenberg* orthobunyavirus | 1256 | 6196 | 299 | 2203 | 932 |
| *Phasmaviridae* | *Orthophasmavirus* barstukasense | 404 | 171 | 534 | 55462 | 239 |
| *Phenuiviridae* | *Phasi* Charoen-like phasivirus | 309471 | 9537924 | 3973106 | 7968902 | 597046 |
| *Phenuiviridae* | *Aphis* citricidus bunyavirus | 18 | 86 | 0 | 32 | 12 |
| *Phycodnaviridae* | *Chrysochromulina* ericina virus | 78 | 43 | 396 | 102 | 89 |
| *Polydnaviriformidae* | *Bracoviriform* facetosae | 373 | 325 | 371 | 228 | 365 |
| *Potyviridae* | *Noni* mosaic virus | 103 | 72 | 92 | 96 | 70 |
| *Poxviridae* | *BeAn* 58058 virus | 17 | 156 | 0 | 31 | 68 |
| *Sedoreoviridae* | *Liao* ning virus | 447 | 295 | 281 | 261 | 313 |
| *Steitzviridae* | *Gihfavirus* pelohabitans | 27341 | 124273 | 8136 | 39198 | 28013 |
| *Steitzviridae* | *Kinglevirus* lutadaptatum | 3937 | 12685 | 1102 | 6574 | 2705 |
| *Straboviridae* | *Dhakavirus* bp7 | 15795 | 83644 | 3737 | 28087 | 12395 |
| *Straboviridae* | *Gualtarvirus* mp1 | 22 | 24 | 12 | 926 | 18 |
| *Tobaniviridae* | *Torovirus* banli | 362 | 1641 | 95 | 504 | 275 |
| Unclassified viruses | *Escherichia* virus DE3 | 9016 | 41589 | 1880 | 14340 | 6533 |
| Unclassified viruses | *Biseptimavirus* P1105 | 1306 | 6614 | 511 | 2552 | 1150 |
| Unclassified viruses | *Punavirus* P1 | 891 | 4506 | 124 | 1628 | 568 |
| Unclassified viruses | *Shinobi* tetravirus | 370 | 114 | 249 | 1393763 | 234 |
| Unclassified viruses | *Traversvirus* P27 | 234 | 1032 | 68 | 376 | 179 |
| Unclassified viruses | *Wenzhou* sobemo-like virus 4 | 186 | 114 | 184 | 490006 | 143 |
| Unclassified viruses | *Pseudomonas* phage phiAH14a | 119 | 632 | 28 | 207 | 100 |
| Unclassified viruses | *Shigella* phage SfIV | 104 | 475 | 53 | 112 | 118 |
| Unclassified viruses | *Omarvirus* omar | 59 | 101 | 43 | 59 | 38 |
| Unclassified viruses | *Marfavirus* F48 | 45 | 29 | 74 | 732 | 39 |
| Unclassified viruses | *Pseudomonas* phage YMC12/01/R24 | 42 | 179 | 0 | 54 | 36 |
| Unclassified viruses | *Pseudomonas* phage UFJF PfDIW6 | 31 | 132 | 0 | 43 | 17 |
| Unclassified viruses | *Polybotosvirus* Atuph07 | 18 | 152 | 0 | 48 | 44 |
| Unclassified viruses | *Seunavirus* GAP31 | 17 | 14 | 98 | 38 | 74 |
| Unclassified viruses | *Escherichia* phage D6 | 12 | 43 | 0 | 132 | 18 |
| Unclassified viruses | *Hubei* mosquito virus 2 | 0 | 0 | 0 | 1654 | 0 |
| Unclassified viruses | *Bacillus* phage v B-Bak10 | 0 | 0 | 0 | 117 | 0 |
| Unclassified viruses | *Escherichia* phage HK639 | 0 | 14 | 0 | 103 | 15 |
| Unclassified viruses | *Menderavirus* mendera | 0 | 87 | 0 | 26 | 29 |
| Unclassified viruses | *Streptococcus* phage 20617 | 0 | 670 | 0 | 0 | 0 |
| **Family** | **Species** | **HWBW** | **HTBW** | **DFBW** | **YGHBW** | **WSBW** |
| *Ascoviridae* | *Ascovirus* *TnAV2a* | 1566 | 204 | 630 | 0 | 0 |
| *Autographiviridae* | *Teseptimavirus* *T7* | 2710 | 379 | 1072 | 368 | 14614 |
| *Autographiviridae* | *Teseptimavirus* *YpsPG* | 0 | 0 | 0 | 0 | 293 |
| *Baculoviridae* | *Alphabaculovirus* *lafiscellariae* | 698 | 744 | 1423 | 853 | 553 |
| *Baculoviridae* | *Betabaculovirus* *plinterpunctellae* | 186 | 192 | 478 | 260 | 135 |
| *Baculoviridae* | *Alphabaculovirus* *altersperidanae* | 71 | 99 | 181 | 80 | 46 |
| *Baculoviridae* | *Choristoneura* *fumiferana* *granulovirus* | 343 | 97 | 74 | 61 | 1196 |
| *Baculoviridae* | *Betabaculovirus* *clanastomosis* | 84 | 170 | 0 | 0 | 0 |
| *Chaseviridae* | *Pahsextavirus* *pAh6C* | 160 | 378 | 100 | 185 | 94 |
| *Hantaviridae* | *Oxbow* *orthohantavirus* | 1210 | 215 | 729 | 439 | 732 |
| *Herpesviridae* | *Ovine* *gammaherpesvirus* *2* | 73 | 295 | 105 | 58 | 102 |
| *Inoviridae* | *Inovirus* *M13* | 3155 | 374 | 1050 | 345 | 17809 |
| *Kyanoviridae* | *Lowelvirus* *tuscon4d* | 95 | 211 | 84 | 163 | 55 |
| *Kyanoviridae* | *Synechococcus* *phage* *S-H38* | 0 | 0 | 872 | 11 | 0 |
| *Leisingerviridae* | *Methanothermobacter* *phage* *psiM100* | 52 | 110 | 247 | 110 | 13 |
| *Mimiviridae* | *Moumouvirus* | 120 | 107 | 115 | 247 | 8852 |
| *Mitoviridae* | *Duamitovirus* *bodo1* | 266 | 86 | 198 | 129 | 107 |
| *Peduoviridae* | *Wadgaonvirus* *wv5004651* | 115 | 35 | 411 | 81 | 7322 |
| *Peduoviridae* | *Gemsvirus* *gv5004652* | 76 | 0 | 60 | 22 | 1061 |
| *Peduoviridae* | *Elveevirus* *4LV2017* | 42 | 0 | 0 | 0 | 416 |
| *Peduoviridae* | *Escherichia* *phage* *Cartapus* | 35 | 0 | 41 | 0 | 386 |
| *Peduoviridae* | *Kapieceevirus* *ST512KPC3phi132* | 0 | 0 | 0 | 0 | 225 |
| *Peduoviridae* | *Vimunumvirus* *ST147VIM1phi71* | 0 | 0 | 0 | 0 | 200 |
| *Peribunyaviridae* | *Simbu* *orthobunyavirus* | 3689 | 528 | 1500 | 442 | 20021 |
| *Peribunyaviridae* | *Schmallenberg* *orthobunyavirus* | 2127 | 342 | 862 | 309 | 11322 |
| *Phasmaviridae* | *Orthophasmavirus* *barstukasense* | 281252 | 38619 | 272 | 284 | 3308955 |
| *Phenuiviridae* | *Phasi* *Charoen-like* *phasivirus* | 2916 | 2075 | 2940 | 2835 | 3087 |
| *Phenuiviridae* | *Aphis* *citricidus* *bunyavirus* | 32 | 20 | 14 | 13 | 415 |
| *Polydnaviriformidae* | *Bracoviriform* *facetosae* | 317 | 313 | 313 | 337 | 218 |
| *Rhabdoviridae* | *Vesiculovirus* *piry* | 16 | 35 | 0 | 21 | 176 |
| *Steitzviridae* | *Gihfavirus* *pelohabitans* | 32054 | 8060 | 16993 | 7354 | 178790 |
| *Steitzviridae* | *Kinglevirus* *lutadaptatum* | 5006 | 1728 | 2552 | 1003 | 18082 |
| *Straboviridae* | *Dhakavirus* *bp7* | 20492 | 6608 | 10960 | 3627 | 144807 |
| *Tobaniviridae* | *Torovirus* *banli* | 507 | 84 | 261 | 110 | 2792 |
| *Tospoviridae* | *Pepper* *chlorotic* *spot* *orthotospovirus* | 848 | 0 | 34 | 0 | 883 |
| *Zierdtviridae* | *Corynebacterium* *virus* *Darwin* | 0 | 0 | 0 | 0 | 426 |
| Unclassified viruses | *Marfavirus* *F48* | 1910 | 3249 | 1188 | 1924 | 3047 |
| Unclassified viruses | *Escherichia* *virus* *DE3* | 13623 | 2126 | 5183 | 1795 | 77544 |
| Unclassified viruses | *Biseptimavirus* *P1105* | 2605 | 1139 | 1162 | 566 | 14290 |
| Unclassified viruses | *Shinobi* *tetravirus* | 213 | 134 | 215 | 153 | 155 |
| Unclassified viruses | *Punavirus* *P1* | 1306 | 157 | 395 | 129 | 8943 |
| Unclassified viruses | *Microbacterium* *phage* *Lynlen* | 96 | 161 | 194 | 116 | 79 |
| Unclassified viruses | *Wenzhou* *sobemo-like* *virus* *4* | 181 | 47985 | 141216 | 99 | 1992 |
| Unclassified viruses | *Traversvirus* *P27* | 317 | 68 | 207 | 70 | 1676 |
| Unclassified viruses | *Shigella* *phage* *SfIV* | 202 | 37 | 100 | 40 | 768 |
| Unclassified viruses | *Pseudomonas* *phage* *phiAH14a* | 147 | 20 | 65 | 17 | 796 |
| Unclassified viruses | *Myosmarvirus* *MTx* | 0 | 0 | 0 | 0 | 687 |
| Unclassified viruses | *Pseudomonas* *phage* *YMC12/01/R24* | 31 | 0 | 15 | 0 | 228 |
| Unclassified viruses | *Polybotosvirus* *Atuph07* | 52 | 15 | 127 | 0 | 185 |
| Unclassified viruses | *Hubei* *mosquito* *virus* *2* | 0 | 138 | 205 | 0 | 0 |
